# Cross-Sectional Study to Evaluate Disparity in Healthcare Access for Patients With a Headache Having Cigna or Medicaid Insurance

**DOI:** 10.7759/cureus.63275

**Published:** 2024-06-27

**Authors:** Valentyna Olinchuk, Souwdamini Sethuram, Adik Umeshkumar Patel, Nadia Djahanshahi, Samreen Shaikh, Naga Amrutha Varshini Nathani

**Affiliations:** 1 Internal Medicine, Bukovinian State Medical University, Chernivtsi, UKR; 2 Internal Medicine, Thanjavur Medical College, Thanjavur, IND; 3 Family Medicine, Texila American University, Providence, GUY; 4 Internal Medicine, Saint George’s University School of Medicine, True blue, GRD; 5 Internal Medicine, Ivane Javakhishvili Tbilisi State University, Tbilisi, GEO; 6 Internal Medicine, NRI Medical College, Mangalagiri, IND

**Keywords:** usa, headache, cigna, medicaid, waiting times, health insurance, healthcare access disparity

## Abstract

Introduction: This study aims to study the disparity in Cigna and Medicaid insurance holders, to secure an appointment for a patient with a headache for two days unrelieved by over-the-counter medication.

Methodology: This is a cross-sectional “secret shopper” type study, assessing the three most populated cities in seven states with the lowest Medicaid coverage and Internal Medicine specialists within a 10-mile radius, with a minimum rating of 3 stars and a willingness to accept new patients.

Results: There was a statistically significant difference in the average waiting period for those with Medicaid and Cigna in the states of Missouri, Nebraska, and Utah, as well as the total average for all seven states. Moreover, there were more healthcare providers who accepted Medicaid rather than Cigna in New Hampshire; whereas in Wyoming, the numbers for Medicaid and Cigna were almost equal.

Conclusions: The significant Medicaid-Cigna acceptance rate disparities should be corrected to ensure higher healthcare access.

## Introduction

Disparities in access to medical services persist as a critical concern, impacting various patient populations. This cross-sectional study aims to analyze Medicaid and Cigna insurance providers and their impact on the timeliness and adequacy of medical interventions for headache patients. Headaches, often dismissed as mundane afflictions, have the potential for profound distress if not promptly addressed. They can often be indicative of underlying health conditions and demand urgent medical attention to mitigate potential complications [1].

Our research recognizes the urgency of addressing healthcare access patterns concerning headache patients based on their insurance coverage [2]. Health insurance plays a pivotal role in the healthcare system, serving as a crucial mechanism for individuals to access medical services and manage the financial costs associated with healthcare [3]. The different types include private health insurance, exemplified by companies like Blue Cross Blue Shield and Cigna, which allow individuals to purchase plans with diverse coverage options. Employer-sponsored insurance (ESI) is provided by employers, often covering a portion of premium costs for employees. Government-sponsored programs include Medicare for individuals aged 65 and older and Medicaid for low-income individuals. Affordable Care Act (ACA) Marketplace Plans, such as those on Healthcare.gov, offer standardized coverage options with subsidies [4]. Each type contributes to the intricate landscape of healthcare access, influencing coverage, costs, and provider networks.

Existing research by Trivedi and Ayanian from 2006 and many others consistently indicates significantly elevated instances of insurance-based discrimination among individuals with public insurance compared to those with private insurance [5]. This establishes a correlation between reported incidents of insurance-based discrimination and adverse healthcare outcomes, including delays and avoidance of necessary care, diminished confidence in accessing needed services, and accounts of subpar care quality [5-7].

By analyzing healthcare access patterns based on insurance types, our research aims to uncover disparities that might contribute to delays in diagnosis, inadequate treatment, and overall suboptimal outcomes for headache patients. Policymakers and healthcare institutions can leverage these insights to develop targeted strategies that address the unique challenges posed by insurance-based discrimination within the Medicaid and Cigna landscapes, ultimately contributing to a more equitable healthcare system.

Thus, the present cross-sectional study seeks to assess and compare healthcare access disparities for patients with headaches between Medicaid and Cigna insurance providers. The primary objective is to investigate potential variations in access to headache treatment services and identify contributing factors to healthcare disparities within these two insurance programs. Additionally, the study aims to analyze and evaluate how waiting times for headache treatment services vary across different states for patients covered by both Medicaid and Cigna. The findings from this research will contribute to a comprehensive understanding of healthcare access challenges, informing targeted interventions and policy recommendations to address state-specific variations in waiting times for headache care.

## Materials and methods

This is a cross-sectional “secret shopper” study, conducted virtually in a week from October 30th to November 6th, 2023 where participants made simulated patient calls to healthcare providers in different parts of the USA [8]. A useful strategy to identify barriers to medical professional appointments is the use of secret shopper protocols, which mimic real-world circumstances for patients [8]. Due to the absence of direct patient-human interaction, ethics approval was exempted [9].

The study focused on headaches as a symptom and the researchers used a case scenario involving a “26-year-old individual experiencing headaches for two days, not relieved by Tylenol (Acetaminophen)”. Researchers then contacted healthcare providers’ offices in the state and city assigned, explained the scenario, and enquired about acceptance of Medicaid and Cigna insurance, and the typical wait time for an appointment according to the insurance. Healthcare providers were chosen from the three most populous cities in states with the lowest Medicaid coverage, which were identified using government websites such as HealthCare.gov and Medicaid Fact Sheets [10,11]. The seven selected states with the lowest Medicaid coverage comprised Wyoming, Utah, Nebraska, Missouri, New Hampshire, North Dakota, and South Dakota. The three most populated cities within each state mentioned above were sourced from Demographics.com (e.g., Wyoming- Cheyenne, Gillette, and Casper). To assess the availability of healthcare professionals, information from each city was collected using HealthGrades. This strategy attempted to examine healthcare professionals’ clinic’s responses to headache-related scenarios in states and major cities with limited Medicaid coverage, providing valuable insights into the accessibility and responsiveness of healthcare services.

To structure the study effectively, inclusion and exclusion criteria were established for healthcare providers. The inclusion criteria specify Internal Medicine specialists within a 10-mile radius, with a minimum rating of 3 stars and a willingness to accept new patients. Exclusion criteria were applied to retired practitioners, permanently closed, or unwilling to accept new patients, and those with incorrect or unreachable contact information via cellphone call. The researchers avoided booking or confirming appointments to ensure resource efficiency and prevent interference with authentic patient appointments. In cases where initial contact with the office proved challenging, such as being directed to voicemail or enduring hold times exceeding 10 minutes, the researchers attempted a recall on a different date. Practitioners’ exclusion from the study was warranted if their office could not be reached on two separate occasions due to poor contact.

Each healthcare practitioner’s profile included documented parameters such as gender (identified through photographs from the website), professional designation, and location specified by state and city. The healthcare professionals were also categorized based on their acceptance of Medicaid and Cigna health insurance, providing valuable insights into insurance preferences within the medical community. Furthermore, healthcare practitioners were also classified based on the waiting time required for the next available appointments depending on insurance and their current willingness to accept new patients under specific insurance coverage. The data was collected via Microsoft Excel. Statistical analysis was performed using StataCorp. 2023. Pearson’s chi-square test was used to calculate the p-value.

## Results

**Figure 1:**
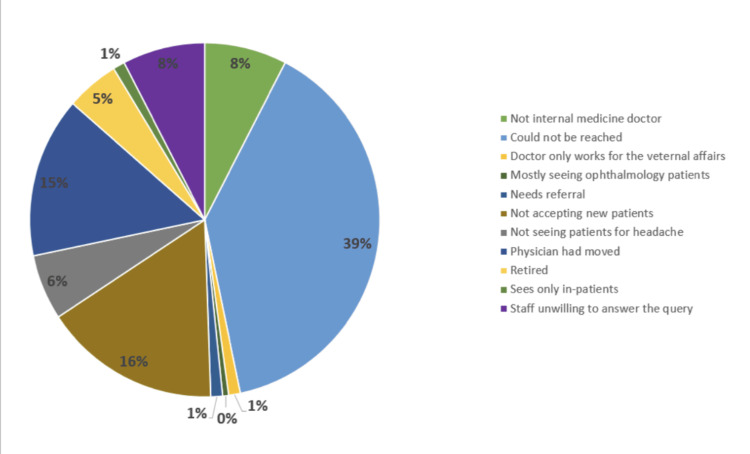
Reasons for Exclusion of Healthcare Providers From the Study The image is drawn by the authors of this article.

At the end of the data collection, a total of 338 physicians from seven states were screened, of which 184 were excluded, with 154 being included in the study. Figure 1 shows the reasons for the exclusion of healthcare providers from the study, wherein they could not be reached (39%), were not accepting new patients (16%), or had moved (15%).

Table 1 shows the characteristics of 154 healthcare providers with the majority being male 60.39% (n=93). The physicians who had an MD accounted for 94.81% (n=148). Those with a 5-star and 4-star ratings were found in 31.17% and 44.16% of the physicians, respectively. We analyzed the data of physicians residing in seven states, of which Nebraska (24.03%), Utah (22.73%), and New Hampshire (17.53%) had the greatest number of physicians included in the study. 

**Table 1:** Characteristics of Healthcare Providers APRN: Advanced Practice Registered Nurses; DO: Doctor of Osteopathic Medicine; MD: Doctor of Medicine Values are written in n (%).

Variables	N (%)
Gender	
Male	93 (60.39)
Female	61 (39.61)
Designation	
MD	146 (94.81)
DO	5 (3.25)
APRN	3 (1.95)
Star/Rating	
5 stars	48 (31.17)
4 stars	68 (44.16)
3 stars	38 (24.68)
State	
Missouri	4 (2.60)
Nebraska	37 (24.03)
New Hampshire	27 (17.53)
North Dakota	21 (13.64)
South Dakota	11 (7.14)
Utah	35 (22.73)
Wyoming	19 (12.34)

As shown in Table 2, there were more healthcare providers who accepted Medicaid (24.49%) rather than Cigna (16.42%) in New Hampshire (P= 0.02). Whereas in Wyoming, the numbers for Medicaid (14.29) and Cigna (10.45) were almost equal (P=0.001), indicating that in the states of New Hampshire and Wyoming, there existed a statistically significant difference between the number of healthcare providers who accepted Medicaid or Cigna as insurance.

**Table 2:** Healthcare Providers Accepting Medicaid and Cigna Values are mentioned in n (%). Test used: Pearson’s chi-square test. P-value <0.05 is considered significant. *P-values that were found to be statistically significant, highlighting those values that were relevant to our study.

State	Medicaid	Cigna	P-value
Missouri	0	4 (2.99)	-
Nebraska	20 (20.41)	35 (26.12)	0.11
New Hampshire	24 (24.49)	22 (16.42)	0.02*
North Dakota	9 (9.18)	21 (15.67)	-
South Dakota	6 (6.12)	10 (7.46)	0.33
Utah	25 (25.51)	28 (20.90)	0.10
Wyoming	14 (14.29)	14 (10.45)	0.001*
Total	98	134	0.17

Table 3 outlines the average waiting period (in days) stratified according to the type of insurance, into Medicaid (n=98) or Cigna (n=134). It further revealed that patients with Cigna had a longer average waiting period to see a healthcare provider in Missouri (1 ± 0.81 vs. 0, P=0.001), Nebraska (10.91 ± 15.67 vs. 4.54± 11.48, P=0.04) and Utah (9.31 ± 15.11 vs. 9.08 ± 15.63, P=0.009) than for those with Medicaid. The study also showed that the total average waiting period for patients in all seven states was longer for those with Cigna than Medicaid (21.66 ± 34.57 vs. 20 ± 35.79, P=0.001). These results suggest that there was a statistically significant difference in the average waiting period for those with Medicaid and Cigna in the states of Missouri, Nebraska and Utah, as well as the total average for all seven states.

**Table 3:** Average Waiting Days Based on Type of Insurance Values are mentioned as mean ± standard deviation. Test used: Kruskal-Wallis test. P-value <0.05 is considered significant. *P-values that were found to be statistically significant, highlighting those values that were relevant to our study.

State	Medicaid (n=98)	Cigna (n=134)	P-value
Missouri	0	1 ± 0.81	0.001*
Nebraska	4.54± 11.48	10.91 ± 15.67	0.04*
New Hampshire	67.11 ± 51.05	60.81 ± 53.40	0.51
North Dakota	2 ± 2.64	2.81 ± 1.72	0.10
South Dakota	22.5 ± 21.21	32 ± 15.67	0.11
Utah	9.08 ± 15.63	9.31 ± 15.11	0.009*
Wyoming	26.31 ± 37.98	29.47± 38.16	0.07
Total	20 ± 35.79	21.66 ± 34.57	0.001*

## Discussion

The key role of this study was to assess the disparity in healthcare access based on insurance type for patients with headaches. This study evaluated waiting times based on Medicaid and Cigna insurance providers at healthcare professionals in seven states: Missouri, Nebraska, New Hampshire, North Dakota, South Dakota, Utah, and Wyoming.

There are two main types of health insurance across the USA - Medicaid and cost-sharing and private insurance. Medicaid, the public insurance program that covers 58 million low-income Americans, improves health outcomes and access to care for its beneficiaries [12]. Although Medicaid improves access to care, specialist care is often unattainable because the program pays low fees to physicians, who are free to turn away Medicaid patients [13]. Cost-sharing and private insurance plans are more advanced and expensive types of insurance. There are many options on how you can purchase them. For example, through the employer or federal healthcare Marketplace. In 2002, the Centers for Medicare and Medicaid Services implemented a 5.4% cut in Medicare physician payments. These cuts have raised concerns that beneficiaries of Medicare will face significant problems obtaining needed physician services [14]. Doctors may choose to stop accepting specific insurance plans based on factors such as reimbursement rates and the administrative burden linked to processing insurance claims.

Table 2 illustrates that in New Hampshire, a higher percentage of healthcare providers accepted Medicaid compared to Cigna, with a statistically significant difference. Conversely, in Wyoming, the acceptance rates for Medicaid and Cigna were nearly equal and were found to have a significant difference as well. The results highlight a statistically significant distinction in the acceptance of Medicaid or Cigna insurance among healthcare providers in these states. In 2017, a similar study was performed, the objective of which was to compare rates of obtaining eye care appointments and appointment wait time for those with Medicaid vs. those with private insurance [15]. This study found that adults and children with Medicaid were less successful in making eye care appointments than those with private insurance. The most commonly provided reason for not offering appointments to patients with Medicaid was that the practice did not accept their insurance [15]. Previous studies have found that lower socioeconomic status is associated with decreased healthcare use [15], including preventive disease screening [16], and worse health outcomes [17,18]. The doctor’s appointment waiting time is highly variable across the US and mostly depends on certain physician specialties as well as a geographical location across the country.

Patients with Cigna experienced a longer average waiting period to see a healthcare provider in Missouri, Nebraska, and Utah compared to those with Medicaid. The study also demonstrated that the overall average waiting period for patients across all seven states was longer for Cigna than for Medicaid. These results highlight a statistically significant difference in average waiting periods between Medicaid and Cigna in Missouri, Nebraska, and Utah, and the overall average for all seven states. A similar study was conducted to analyze appointment access and wait times for dermatologists [14] which found that in communities with relatively low Medicaid payment rates, patients with Medicaid faced higher rejection rates and longer wait times.

This study, as cross-sectional studies do, has some limitations. HealthGrades was the only website used for gathering information of the doctors in the selected cities. Doctors only with rating above 3 stars and within a 10-mile radius were considered. Actual patient experience might differ from the case story. More states could have been included to increase the statistical power of study, whereas only seven states with the lowest Medicaid coverage were selected. Also, this study only includes two insurance providers which limits the knowledge of disparity.

## Conclusions

This cross-sectional study identified significant disparities in healthcare access based on insurance, for patients presenting with headaches in seven US states with the lowest Medicaid coverage. Insurance acceptance varied significantly. New Hampshire had higher Medicaid acceptance compared to Cigna, while Wyoming showed nearly equal acceptance for both. These regional differences in insurance preferences suggest varying impacts on patient access to care. Patients with Cigna generally experienced longer waiting periods for appointments compared to those with Medicaid, especially in Missouri, Nebraska, and Utah. This indicates potential insurance-based discrimination in appointment scheduling, affecting timely healthcare access. This study highlights the need for policy interventions to improve healthcare accessibility and equity. Addressing identified barriers can enhance service delivery and ensure timely, effective medical care for all patients, regardless of insurance status. Future research should explore the causes of these disparities and develop strategies to mitigate them, promoting a more inclusive healthcare environment.

## References

[1] Takeshima T, Kikui S (2013). Special Feature: Common diseases are the main battlefield of neurology - current situation and prospects. (Article in Japanese). Brain Nerve.

[2] Shao Q, Rascati KL, Barner JC, Lawson KA, Sonawane KB, Rousseau JF (2022). Healthcare utilization and costs among patients with chronic migraine, episodic migraine, and tension-type headache enrolled in commercial insurance plans. Headache.

[3] Griffith KN, Jones DK, Bor JH, Sommers BD (2020). Changes in health insurance coverage, access to care, and income-based disparities among us adults, 2011-17. Health Aff.

[4] Donohue JM, Cole ES, James CV, Jarlenski M, Michener JD, Roberts ET (2022). The US Medicaid program: Coverage, financing, reforms, and implications for health equity. JAMA.

[5] Trivedi AN, Ayanian JZ (2006). Perceived discrimination and use of preventive health services. J Gen Intern Med.

[6] Han X, Call KT, Pintor JK, Alarcon-Espinoza G, Simon AB (2015). Reports of insurance-based discrimination in health care and its association with access to care. Am J Public Health.

[7] Allen H, Wright BJ, Harding K, Broffman L (2014). The role of stigma in access to health care for the poor. Milbank Q.

[8] Fryer K, Reid CN, Elmore AL (2023). Access to prenatal care among patients with opioid use disorder in Florida: Findings from a secret shopper study. Obstet Gynecol.

[9] Kolesova M, Sarantos S, Alvarez J, Torres A, Pateriya S, Penalver M (2023). Accessibility to obstetric care in South Florida based on insurance: A cross-sectional study. Cureus.

[10] (2024). Medicaid & CHIP Coverage. https://www.healthcare.gov/medicaid-chip/.

[11] (2024). Medicaid State Fact Sheets. https://www.kff.org/interactive/medicaid-state-fact-sheets/.

[12] Sommers BD, Baicker K, Epstein AM (2012). Mortality and access to care among adults after state Medicaid expansions. N Engl J Med.

[13] Rosenbaum S (2014). Medicaid payments and access to care. N Engl J Med.

[14] Resneck Jr J, Pletcher M, Loranzo N (2004). Medicare, Medicaid, and access to dermatologists: The effect of patient insurance on appointment access and wait times. J Am Acad Dermatol.

[15] Lee YH, Chen AX, Varadaraj V (2018). Comparison of access to eye care appointments between patients with Medicaid and those with private health care insurance. JAMA Ophthalmol.

[16] Kim MJ, Lee H, Kim EH, Cho MH, Shin DW, Yun JM, Shin JH (2017). Disparity in health screening and health utilization according to economic status. Korean J Fam Med.

[17] Griffith K, Evans L, Bor J (2017). The affordable care act reduced socioeconomic disparities in health care access. Health Aff.

[18] Rohlfing ML, Mays AC, Isom S, Waltonen JD (2017). Insurance status as a predictor of mortality in patients undergoing head and neck cancer surgery. Laryngoscope.

